# Psychopathological Aspects in Childhood Hematopoietic Stem Cell Transplantation (HSCT): The Perception of Parents and Adolescents

**DOI:** 10.3389/fpsyg.2017.00272

**Published:** 2017-04-05

**Authors:** Silvia Zanato, Annalisa Traverso, Marta Tremolada, Francesco Sinatora, Alessio Porreca, Giorgio Pozziani, Nicoletta Di Florio, Fabia Capello, Antonio Marzollo, Manuela Tumino, Chiara Cattelan, Giuseppe Basso, Chiara Messina

**Affiliations:** ^1^Psychiatric Unit, Department of Women's and Children's Health, University Hospital of PaduaPadua, Italy; ^2^Department of Developmental and Social Psychology, University of PaduaPadua, Italy; ^3^Haematology-Oncology Division, Department of Women's and Children's Health, University Hospital of PaduaPadua, Italy

**Keywords:** HSCT, transplantation, childhood HSCT survivors, HSCT psychological sequelae, child psychopathology, adolescent psychopathology

## Abstract

**Background:** Data about psychosocial sequelae of childhood Hematopoietic Stem Cell Transplantation (HSCT) are limited and the association with a specific donor type or other medical factors is largely unknown (Chang et al., 2012). The aim of the present study was to compare the psychological aspects of pediatric HSCT survivors with healthy peers. A secondary aim was to detect whether parents and children differed in the perception of mental health status. The influence of medical factors on psychological status was also examined.

**Method:** Thirty seven HSCT survivors (23 males) with a mean age of 14.4 years (*SD* = 3.03; range 8.16–18.33) were recruited. Twenty-six patients underwent an allogenic HSCT (matched unrelated donor, *n* = 20; matched sibling donor, *n* = 6) and 11 patients received an autologous HSCT. The children psychological aspects were assessed using the Youth Self Report (YSR) (Achenbach and Rescorla, 2001) and compared to a group of matched healthy peers. At the same time, parents were requested to complete the Child Behavior Checklist 6–18 (Achenbach and Rescorla, 2001). Medical and socio-demographic data were also collected.

**Results:** HSCT survivors reported significantly higher levels of somatic complains (*t*_27_ = 3.14; *p* = 0.004; mean = 3.1) when compared to healthy peers (mean = 1.5). The parent CBCL scores on “child total competence” exceeded the normative clinical cutoff in 48.6% cases. Inter-rater agreement between parent and patient reports was present only in three scales: total competence score (*K* = 0.06, *p* = 0.002), somatic complaints (*K* = 0.21, *p* = 0.003) and attention problems (*k* = 0.13; *p* = 0.02). According to Ancova models, internalizing problems were more frequent in HSCT from family donors (*F*_2_ = 3.13; *p* = 0.06) or in the presence of acute complications (*F*_1_ = 11.95; *p* = 0.003).

**Conclusion:** In contrast to the perception of parents, pediatric HSCT survivors reported good psychological health. However, they complained about more somatic problems as compared with healthy peers. Medical aspects such as donor source and the presence of acute complications should be taken into consideration for the psychological approach in order to improve pediatric HSCT survivor care.

## Introduction

Hematopoietic Stem Cell Transplantation (HSCT) is a medical procedure involving the collection and re-infusion of multipotent hematopoietic stems cells, aimed at the reconstitution of a normal hematopoietic function (Edman et al., 2001; DeMarinis et al., 2009). Stem cells can be derived from the patient themselves (autologous HSCT) or from a donor (allogenic HSCT). These procedures differ both in indication and in the patient population involved. Autologous HSCT is generally performed to rescue from a dose limiting myelotoxicity patient undergoing chemotherapy for solid tumors or multiple myeloma. Allogenic HSCT is aimed at replacing partially or completely the hematopoietic stem cell compartment with cells derived from a donor, in order to cure hematologic malignancies, non-malignant hematologic disorders, primary immunodeficiency diseases, and inborn errors of metabolism. The donor from allogenic HSCT may be a family member or an unrelated volunteer; stem cells may be derived from bone marrow, umbilical cord blood, or peripheral blood after treatment with a mobilizing agent (Park et al., 2015).

More than 50,000 HSCTs are performed every year worldwide and around 5,000 of them every year in Italy (Gratwohl et al., 2010). The process of care for a patient undergoing HSCT involves different phases: preparatory evaluation, high-doses chemotherapy, stem cell infusion, aplasia, marrow recovery, and a life-long follow-up. Although outcomes have improved over the years, patients are at risk of early and late side effects that range from mild to life-threatening events (Sanders, 2002; Tabbara et al., 2002; Andersson et al., 2008). Early complications include regimen-related toxicity, bleeding, and infections (Whedon and Wujcik, 1997). In the setting of allogenic HSCT, Graft-vs.-Host disease (GvHD), an immune reaction of the donor lymphocytes against the recipient tissue, may lead to significant morbidity and mortality. Late effects of HSCT may impact on the function of different organs resulting in chronic cardiovascular, respiratory, metabolic, endocrine, and reproductive disorders (Sanders, 2002; Tabbara et al., 2002).

About 17% of HSCTs are performed in children under 20 years of age (Center for International Blood and Marrow Transplant Research, 2005; Hollingsworth et al., 2008) and particular considerations should be applied to HSCT at this age (Hollingsworth et al., 2008; Chang et al., 2012). Despite its increasing success rates, pediatric HSCT still carries a significant risk of emotional, cognitive, social, and family functioning (Clarke et al., 2008). The aversive experiences and life disruptions experienced by pediatric patients undergoing transplantation can interfere with adjustment and consequently with social and emotional maturation (Vannatta et al., 1998). Survivors have been described as being more withdrawn and less socially competent with respect to their peers (Phipps and Mulhern, 1995; Vannatta et al., 1998). Moreover, given the longer life expectancy of childhood HSCT survivors as compared with adults, different medical complications, such as GvHD, endocrine dysfunctions and pulmonary complications might arise (Buchsel et al., 1996). Although empirical data about the psychological consequences of HSCT during pediatric age are still limited, there is some evidence that children and adolescents that undergo HSCT, as well as their families, are exposed to intense emotional distress and to multiple traumatic situations (Packman et al., 2010). Experiencing disease during childhood constitutes a risk factor for abnormal personality development: given the close relationship between body and mind, especially during childhood and adolescence, each physical intervention could cause trauma in psychological functioning. Moreover, receiving a cancer diagnosis in adolescence may impact on processes such as physical and sexual maturation, development of identity, and the possibility to experience independence, decision making and social growth (Geenen et al., 2007; Kieran et al., 2010; Kahalley et al., 2013). Even though HSCT offers higher life expectancies, it is associated with intense emotional distress for patients and their families, might cause trauma and could affect the possibility to re-establish normal life routines (Panizon, 2005). More than a third of childhood cancer survivors show physical, neurocognitive, or psychological sequelae, with subsequent long-term psychopathological outcomes (Institute of Medicine (US) and National Research Council (US) National Cancer Policy Board, 2003).

HSCT survivors are at higher risk of developing mood disorders and anxiety disorders when compared to the general population and to individuals with chronic disorders (Felder-Puig et al., 2006; Packman et al., 2010).

Several studies reported that a consistent amount of patients frequently experiences depression, peer isolation and behavior problems during the 6 months post-HSCT (Pot-Mees, 1989; Ahomäki et al., 2015). Worse depressive symptoms resulted associated with extended hospital stays (Patenaude, 1990) whereas more severe withdrawal and a higher loss of adaptive skills were associated with younger age at transplantation (Pot-Mees and Zeitlin, 1987).

As far as it concerns anxiety, the ongoing of symptoms seems to assume a different direction: a high rate of pediatric patients experiences intense anxiety before HSCT, whereas symptoms are likely to decrease after transplantation and to remain low during follow-up visits (Meyers et al., 1994). Moreover, some studies reported lower degrees of anxiety in younger children, whereas older children and adolescents reported higher anxiety (Nuss and Wilson, 2007).

Besides mood disorders and anxiety disorders, researches have also detected the presence of social anxiety, post-traumatic stress disorders, low levels of self-esteem, and poor quality of life among this population (Stuber et al., 1990; Bessel, 2001; Kazak et al., 2001; Stam et al., 2006). In line with these results, some authors reported that a high percentage of patients show moderate post-traumatic stress symptoms immediately after HSCT (Stuber et al., 1990) and suggested that some of them experience these symptoms even 12 months after HSCT (Pot-Mees, 1989).

Furthermore, although some experiences report good resilience in cancer survivors, others suggested that at least 10–30% of these children is likely to exhibit difficulties in the adjustment subsequent to treatments.

The main risk factors that were associated with these outcomes were: type of disease, higher age at communication of diagnosis, and the adverse effects of the therapies (Levi, 2006; McDougall and Tsonis, 2009). A recent Finnish study compared a cohort of nearly 14,000 cancer survivors with a sample of healthy siblings, finding a higher incidence of psychiatric disorders such as: mood disorders, anxiety disorders, psychotic disorders, somatic disorders, feeding disorders, and personality disorders (Ahomäki et al., 2015). The same study identified radiotherapy, female gender and younger age at diagnosis as principal risk factors. Moreover, other studies reported the type of transplantation to be another clinical variable with an impact on psychological well-being of children and adolescents exposed to HSCT (Phipps et al., 2002).

Although different studies evidenced different HSCT-related psychological stressors, the existing literature still appears scarce. Moreover, it is plausible that, in the context of health care, treatments may not focus specifically on the psychological aspects involved in transplantation but rather consider physical issues and medical complaints (Feeny et al., 1999; Tanzi, 2011). A deeper understanding of the psychological impact of HSCT could help to implement treatment with subsequent improvements in the general Quality of Life (Tanzi, 2011). Moreover, screening ex-patients' emotional and behavioral functioning, and understanding the potential risk factors associated with long-term psychopathological outcomes, could help to inform interventions during recovery, improving the survivors' experience and preventing unnecessary distress.

### Aims and hypothesis

The objective of the present study was to investigate psychopathological aspects in ex-patients that underwent HSCT during childhood, according to both their own perception and the perception of their parents. More specifically, the aims of the study were to: (a) assess the presence of psychopathology in HSCT survivors at distance from transplantation; (b) compare the psychological aspects of pediatric HSCT survivors with the ones of healthy peers; (c) detect whether parents and children differed in their perception of mental health status; (d) identify medical and socio-demographic factors associated with psychopathology.

According to literature, it has been supposed that HSCT survivors present higher levels of psychological distress compared with healthy peers even many years after the experience of transplantation. It is hypothesized that parents perceive less stress and a better quality of life than patients themselves.

## Materials and methods

### Subjects and procedure

The research involved subjects of pediatric age that had undergone HSCT at least 5 years before the recruitment phase (a range of time pointed out by the literature as adequate to consider the subjects as “survivors”) and their families (HSCT Group), and a comparison group of healthy peers (HEALTH Group). The inclusion criteria for the HSCT Group were:

-being <18 years old;
-being an ex-patient that had undergone HSCT at least 5 years previously;
-absence of intellectual disability;
-a stable health condition.

From 2012 to 2014, 77 families were drawn from the HSCT database of the Laboratory of Pediatric Onco-Hematology at the University Hospital of Padua. The families were subsequently recruited through a preliminary phone contact aimed at illustrating the objectives of the study. Families that agreed to participate in the research were sent a folder containing a brief description of the study, informed consent and self-report questionnaires. Subsequently, psychological consultations were carried out during medical post-transplant follow-up visits.

The present study is part of a broader study with the main aim of exploring every aspect of HSCT survivors' lives, including mental health and Quality of Life, using specific psychopathological inventories or questionnaires. Data about the Quality of Life perceived by patients and their parents will be the subject of future reports.

The HSCT Group was matched to the HEALTH Group according to age, gender and geographic origin. The HEALTH Group (M:F = 22:12) was composed of unselected adolescents aged 15–25 years (*M* = 15.07, *SD* = 2.36) recruited in schools, youth groups and universities, through a project aimed at assessing their psycho-social well-being. In order to be included in the HEALTH Group the subjects should not have had experiences of intense hospitalizations or chronic disease. After receiving informed consent, questionnaires were completed online. Written informed consent was obtained from all the parents, whereas all the minors gave their verbal informed consent.

The present study was approved by the Ethical Committee of the Department of Women's and Children's Health of the University Hospital of Padua in July 2013.

### Tools

#### Semi-structured interview and checklist for the collection of case history

All the relevant demographic, medical, and psychological information was investigated using a semi-structured *ad hoc* interview accompanied by a checklist. More specifically, as far as it regards socio-demographic information, the interview investigated: age, gender, and education of the child/adolescent, family composition, socioeconomic status, immigration history, distance between the residence and the medical center. The medical variables^1^ included: underlying disease, length of the isolation associated with the transplantation, and presence of involution. The psychological variables included: spontaneous request for psychological help and presence of previous psychological counseling.

Moreover, the interview and the checklist investigated aspects such as the perinatal period, presence of previous feeding or sleep problems, psychomotor and language development, sphincter control, regularity of menstruation and bodily functions, and specific school difficulties.

This investigation was carried out in order to exclude the possibility that previous psychopathological conditions could influence the current psychological/psychopathological aspects reported and, thus, prevent a correct interpretation of the data collected. The domains investigated were grouped into a unique category (Psychophysical problems) that has been subsequently put in relation with the results of self-report measures.

#### Achenbach's child behavior checklist (CBCL) and youth self-report (YSR)

The Achenbach System of Empirically Based Assessment was developed within a theoretical and empirical paradigm aimed at collecting information regarding adjustment, competence, and emotional/behavioral problems in children and adolescents, emerging in different situations, and collected through multiple informants (a). This latter quality allows the system, based on a multi-axial approach, to reach a measurable global frame of the individual. The assessment groups the identified problems into syndrome scales which can be compared to standardized normative values matched for age and gender. For the purpose of the present study the participants were given the Child Behavior Checklist/6–18 (CBCL—Achenbach and Rescorla, 2001), filled in by the parents, and the Youth Self-Report/11–18 (YSR—Achenbach and Rescorla, 2001).

### The child behavior checklist

The CBCL/6–18 (Achenbach and Rescorla, 2001) is a questionnaire filled in by parents and caregivers with the aim of assessing the child/adolescent's behavioral/emotional functioning. The questionnaire is composed of two parts. The first part investigates Competence and Adaptive behavior (*Activities, Social*, and *School*) whereas the second part investigates the presence of behavioral/emotional problems through 113 items, which can be classified as “normative,” “borderline” or “clinical” according to specific syndrome scales or DSM-Oriented scales. Each item is scored on a 3-point Likert scale (0 = not true, 1 = true in part or sometimes true, 2 = very true, often true) in accordance to the behavior reported. The syndrome scales are: *Anxious/depressed, Withdrawn/Depressed, Somatic Complaints, Social Problems, Thought Problems, Attention Problems, Rule-Breaking Behavior*, and *Aggressive Behavior*. The syndrome scales can be grouped into more general summary scales: *Internalizing Problems, Externalizing problems*, and *Total Problems*. Moreover, the CBCL/6–18 can be scored in terms of DSM-Oriented scales that, although not directly equivalent to a DSM diagnosis, can help to relate standardized, quantified, normed assessment of behavioral/emotional problems to DSM categories. The scales are: *Affective Disorders, Anxiety Disorders, Somatic Problems, Attention Deficit and Hyperactivity Problems, Oppositional Defiant Problems*, and *Conduct Problems*. Finally, the system allows the calculation of scales for *Obsessive-Compulsive Problems, Stress Problems*, and *Sluggish Cognitive Tempo*. For the purpose of the present study Italian normative values where adopted (Frigerio et al., 2006; Ivanova et al., 2007). The CBCL reported excellent reliability (0.85–0.88) (De Groot et al., 1994).

#### The youth self report

The 113-item YSR/11–18 (Achenbach and Rescorla, 2001) was developed as an adapted version of the CBCL for subjects aged 11–18 years old, in order to have a self-report measure on competence and emotional/behavioral problems. The respondent is asked to score the items on a 3-point Likert scale (0 = not true, 1 = in part/sometimes true, 2 = very/often true). As for the CBCL, also in the YSR/11–18 the raw scores lead to 8 syndrome scales (*Anxiety/Depression, Withdrawn/Depressed, Somatic complaints, Social problems, Thought problems, Attention problems, Antisocial behavior, Aggressive behavior*) which can be grouped into summary scales (*Internalizing problems, Externalizing problems, Total problems*). Moreover, the raw scores can be converted into DSM-oriented scales (*Affective problems, Anxiety problems, Somatic problems, Attention problems, Oppositional-defiant problems, Conduct problems, Obsessive-compulsive problems, Post-traumatic stress problems*). The YSR/11–18 has been successfully applied to different cultures and languages, and reported from good to excellent reliability (range = 0.66–0.87; Leung et al., 2006; Ebesutani et al., 2011).

## Results

Data were analyzed using IBM SPSS statistics version 23 [SPSS(R) 23.0] (IBM Corporation, Armonk NY, USA). Descriptive analyses (frequencies, mean scores, standard deviations) were performed on the CBCL and the YSR scores in order to assess the presence of psychopathology. Subsequently, Cohen's kappa coefficient was applied in order to measure inter-rater agreement between the parents and the survivors' reports on psychopathology; for this purpose, contingency tables with the CBCL and the YSR were made. Finally, ANCOVAs were carried out in order to assess the effect of the single independent variables in predicting the presence of psychopathology.

### Descriptive analyses

#### The survivors and their parents

Descriptive analyses (frequencies, mean scores, standard deviations) were run on data concerning the demographic and medical information of the HSCT Group.

Thirty seven families (M: F = 23:14) agreed to participate to the study. Table 1 shows the demographic and medical information of the survivors.

**Table 1:** **Demographic and medical information of the HSCT Group (*N* = 37)**.

	***N*** **(%)**	**M (SD)**	**Range**
**GENDER**			
Male	23 (62%)		
Female	14 (38%)		
Age		15 (3.1)	8.2–18.6
**BASIC PATHOLOGY**			
Leukemia	19 (51%)		
Lymphomas and solid tumors	12 (32%)		
Other^*^	6 (16%)		
**TYPE OF HSCT**			
Autologous	11(30%)		
Allogenic unrelated	20 (54%)		
Allogenic related and/or haploidentical	6 (16%)		
Intensification with double HSCT	4 (11%)		
Age at HSCT		10 (3.4)	0.7–11.2
Time lapsed from HSCT (years.)		9.88 (3.1)	5.7–16.7
Days of hospitalization for HSCT		54.6 (32.5)	21–164
Days of hospitalization after HSCT		13.4 (20.1)	0–91

* *Fanconi anemia, Shwachmann syndrome, aplastic anemia, hemophagocytosis, mucopolysaccharidosis*.

**Figure 1:**
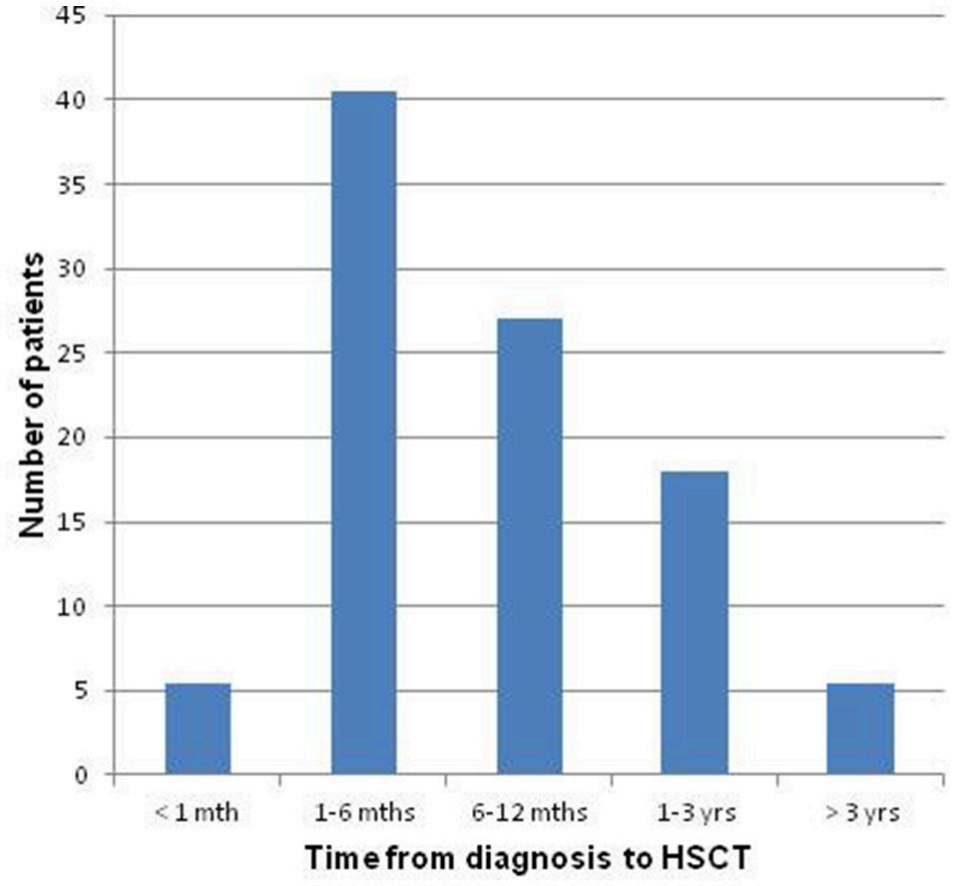
**Time elapsed from diagnosis to effective HSCT**.

**Figure 2:**
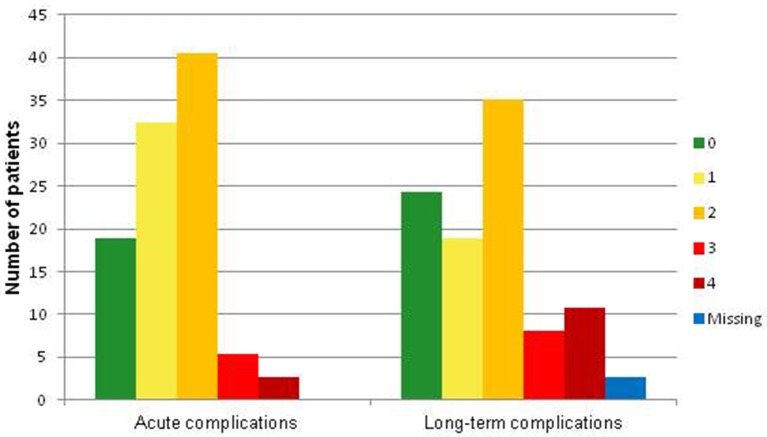
**Frequency of medical complication**.

As can be seen from Figure 1, the time passed from diagnosis to effective transplantation was very heterogeneous among the group. Figure 2 shows the frequencies of severe medical complications. No relevant psychophysical problems were detected among the HSCT group. Regarding survivors' parents, the average age of fathers was 48,6 years (*SD* = 6,0; range 36–61), while for mothers it was 45.0 (*SD* = 5,2; range 34–54). Thirty one couples (84%) were conjugated or cohabiting while in 14% of cases they were separated. In the majority of couples (62%), both parents were employed; in 35% of cases one parent was unemployed, while in only one situation both parents were unemployed.

#### Presence of psychopathology

Descriptive statistics were performed on the results of the CBCL and of the YSR in order to assess the presence of psychopathology in the survivors as referred by the survivors themselves or their parents. The results are shown in Tables 2–4. Tables 2, 3 show the distribution of the subjects within the Normative, the Border, and the Clinical range with respect to the CBCL/6–18 and to the YSR/11–18 scales, whereas Table 4 shows the raw scores of the HSCT and the HEALTH Group to the YSR/11–18.

**Table 2:** Distribution of the subjects within the Normative, the Border, and the Clinical range with respect to the CBCL scales^*^.

	**Norm**		**Border**		**Clinical**	
	***N***	**%**	***N***	**%**	***N***	**%**
**COMPETENCE AND ADAPTIVE BEHAVIOR**						
Activities	16	43.2	11	29.7	8	21.6
Social	26	70.3	4	10.8	4	10.8
School	27	73	2	5.4	4	10.8
Total competence	7	18.9	5	13.5	18	48.6
**SYNDROME SCALES**						
Anxiety/depressive	29	78.4	6	16.2	1	2.7
Depression	30	81.1	3	8.1	3	8.1
Somatic complaints	26	70.3	5	13.5	5	13.5
Social_problems	30	81.1	6	16.2	0	0
Thought	32	86.5	2	5.4	2	5.4
Attention	32	86.5	3	8.1	1	2.7
Rulebreak	35	94.6	1	2.7	0	0
Aggressive	32	86.5	2	5.4	2	5.4
**SUMMARY SCALES**						
Internalizing Pr.	20	54.1	5	13.5	11	29.7
Externalizing Pr.	29	78.4	6	16.2	1	2.7
Total problems	27	73	4	10.8	5	13.5
**DSM-ORIENTED SCALES**						
Anxiety disorders	27	73	7	18.9	2	5.4
Somatic problems	26	70.3	6	16.2	4	10.8
ADHD	35	94.6	1	2.7	0	0
Oppositional	33	89.2	2	5.4	1	2.7
Conduct	34	91.9	2	5.4	0	0
Sluggish_Cogn.	27	73	6	16.2	3	8.1
Obs_Compul	31	83.8	4	10.8	1	2.7
Post_Traum	30	81.1	3	8.1	3	8.1

* *Some data unavailable (not classifiable)*.

**Table 3:** Distribution of the subjects within the Normative, the Border, and the Clinical range with respect to the YSR/11–18 scales^*^.

	**Norm**		**Border**		**Clinical**	
	***N***	**%**	**N**	**%**	***N***	**%**
**COMPETENCE AND ADAPTIVE BEHAVIOR**						
Activities	20	54.1	3	8.1	3	8.1
Social	20	54.1	2	5.4	5	13.5
School	0	0	0	0	0	0
Total_comp	17	45.9	1	2.7	8	21.6
**SYNDROME SCALES**						
Anx/depr	27	73	1	2.7	0	0
Depression	28	75.7	0	0	0	0
Somatic compl	24	64.9	2	5.4	2	5.4
Social_Probl	26	70.3	1	2.7	1	2.7
Thought	28	75.7	0	0	0	0
Attention	28	75.7	0	0	0	0
Rulebreak	28	75.7	0	0	0	0
Aggressive	28	75.7	0	0	0	0
**SUMMARY SCALES**						
Internalizing	23	62.2	3	8.1	2	5.4
Externalizing	28	75.7	0	0	0	0
Total prob	27	73	2	2.7	0	0
**DSM-ORIENTED SCALES**						
Aff_disord	28	75.7	0	0	0	0
Anx_disord	27	73	1	2.7	0	0
Somatic_Pr.	22	59.5	3	8.1	3	8.1
ADHD	28	75.7	0	0	0	0
Opposit	26	70.3	2	5.4	0	0
Conduct	28	75.7	0	0	0	0
Obs_compul	28	75.7	0	0	0	0
Post_traum	28	75.7	0	0	0	0

* *Some data unavailable (not classifiable)*.

**Table 4:** **Mean scores (M) and standard deviations (SD) of the HSCT and the HEALTH Group at the YSR/11–18 with respect to the syndrome scales and the summary scales**.

	**t (df); p**	**HSCT group**		**HEALTH group**	
		***M***	***SD***	***M***	***SD***
**COMPETENCE AND ADAPTIVE BEHAVIOR**					
Activities	1.45 (24); 0.16	9.50	3.09	8.55	2.75
Social	0.26 (25); 0.79	7.69	2.23	7.80	1.94
School	−1.23 (28); 0.23	2.21	0.34	2.32	0.34
Total_Comp	0.65 (24); 0.52	19.07	4.93	18.67	4.00
**SYNDROME SCALES**					
Anx/Depr	−0.30 (28); 0.76	5.00	3.57	5.15	4.36
Depression	0.19 (28); 0.85	3.03	2.26	2.79	2.90
Somatic compl.	2.04 (27); 0.05^*^	4.18	3.00	2.59	2.43
Social_probl	0.5 (27); 0.62	3.54	2.41	3.15	2.63
Thought	−0.4 (27); 0.64	2.79	2.23	2.97	2.52
Attention	−0.89 (27); 0.37	5.11	2.70	5.91	2.80
Rulebreak	−0.9 (27); 0.37	2.11	1.57	2.79	3.42
Aggressive	−0.55 (27); 0.58	6.21	3.53	7.09	3.66
**SUMMARY SCALES**					
Internalizing	0.51 (28); 0.61	12.07	7.44	10.53	8.36
Externalizing	−0.78 (27); 0.44	8.32	4.60	9.88	6.10
Total Problems	0.007 (27); 0.99	36.29	16.57	36.21	19.51

* *Some data unavailable (not classifiable)*.

As it can be seen (Table 2), as far as concerns the domain of competencies, the parents reported values above the clinical range for *Total Competencies* (49%) and *Activities* (22%). Regarding behavioral/emotional problems, on the other hand, the parents seemed to identify more internalizing difficulties, especially regarding *Somatic problems* (11% clinical, 16% border), *Post-traumatic stress disorder* (8% clinical, 8% border), *Sluggish cognitive tempo* (8% clinical, 16% border), *Obsessive-compulsive disorder* (3% clinical, 11% border). According to the parents' perception inherent socialization and externalization difficulties maintained mostly sub-clinical values.

As regards the YSR, as can be seen in Table 3, the major difficulties were detected with respect to *Total competencies* (22% clinical), *Social competencies* (13%) and *Somatic complaints* (8%). The other scales mostly maintained scores within the normative range.

#### Comparison of the YSR in the HSCT group to the health group

The paired sample *T*-test was applied to investigate the presence of differences in the mean scores of the Competence Scales and of the Syndrome Scales between the HSCT Group and the HEALTH Group. Statistically significant differences emerged regarding Somatic symptoms [t_(27)_ = 3.14; *p* = 0.004], which were higher in the HSCT Group compared with the HEALTH Group. No significant differences (*p* > 0.05) were found regarding other variables (Activities, Social, School, Total Competence, Anxiety/Depression, Withdrawn/Depressed, Somatic complaints, Social problems, Thought problems, Attention problems, Antisocial behavior, Aggressive behavior).

#### Inter-rater agreement between parents and survivors in the perception of psychopathology

In order to measure inter-rater agreement between the parents and the survivors' perception of psychopathology Cohen's kappa coefficient was applied; for this purpose, the results of the CBCL and of the YSR were organized into contingency tables. Statistically significant agreement was found for *Total competencies* (*K* = 0.06, *p* = 0.002), *Somatic problems* (*K* = 0.21, *p* = 0.003) and *Attention problems* (*k* = 0.13; *p* = 0.02). The other scales (*Anxiety/Depressive; Depression, Social problems Thought, Rule-break, Aggressive, Internalizing, and Externalizing*) did not show significant agreement (*p* > 0.05). However, basing on Cohen's kappa interpretation by which there is a scarce concordance if kappa assumes values between 0 and 0.4, there was really no overlap between parents' and children's ratings.

#### Factors influencing psychopathology

Preliminarily, correlation analysis, and ANOVAs were run on variables referring to psychopathology and on medical and socio-demographic variables. The distribution of the factors was estimated as normal. In the preliminary Pearson's correlations, the medical variables (i.e., the time between diagnosis and HSCT, medical complications such as GVHD and psycho-physical problems) did not result significantly associated with the psychopathological scales.

Subsequently, in order to investigate whether specific demographic or medical aspects could influence behavioral/emotional problems in HSCT survivors, ANCOVA models were carried out on the CBCL scores (i.e., the psychopathology as perceived by the parents). The dependent variables included: *Internalizing problems, Externalizing problems*, and the *Total problems scale*. The independent variables concerned: type of transplantation (autologous, unrelated donor allogeneic, related donor allogenic), presence of GvHD (no/yes), hospitalization during intensive therapy (no/yes), double intensification of therapy (no/yes), and gender (M/F). As covariates the model included: sum of acute and long-term complications, sum of psychophysical problems, age at transplantation, time elapsed between transplantation and assessment, days of hospitalizations in the Transplantation Unit, time elapsed between the communication of the need to transplant and effective transplantation. ANCOVAs were run also on the CBCL syndrome scales. Table 5 shows the models that were significantly predictive of Internalizing problems, Externalizing problems and the Total problems.

**Table 5:** **Socio-demographic and medical factors highlighted by ANCOVAs as predictors of the CBCL summary scales and the CBCL total score**.

**Dependent variable**	**N**	**IV**	***df***	***F***	***p***	**η_*p*_*2***	***B***	**Estimated marginal means (***SD***)**		
CBCL total problems	37	Type of HSCT	2	10.94	0.004	0.71	0.95	Autologous 43.4 (4.55)	Unrelated donor allogeneic 18.65 (3.91)	Related donor allogeneic 59.5 (5.58)
		Sum of acute complications	1	29.98	0.0001	0.78	1	Mean of covariate Sum of acute complications = 1.39		
		Sum of long term complications	1	9.41	0.01	0.51	0.78	Mean of covariate Sum of long term complications = 1.61		
		Time elapsed between the communication of the HSCT need and the effective HSCT	1	18.58	0.02	0.67	0.97	Mean of covariate Time elapsed between the communication of the HSCT need and the effective HSCT = 177.26		
		Psychophysical problems	1	6.84	0.003	0.43	0.64	Mean of covariate Psychophysical problems = 1.64		
		Gender	1	5.44	0.004	0.37	0.55	Female 46.15 (4.43)		Male 30.82 (3.13)
CBCL internalizing problems	37	Type of HSCT	2	10.94	0.004	0.66	0.89	Autologous 11.80 (1.26)	Unrelated donor allogeneic 8.09 (1.16)	Related donor allogeneic 19.18 (1.63)
		Sum of acute complications	1	25.49	0.001	0.74	0.99	Mean of covariate Sum of acute complications = 1.39		
		Time elapsed between the communication of the HSCT need and the effective HSCT	1	13.27	0.005	0.59	0.9	Mean of covariate Time elapsed between the communication of the HSCT need and the effective HSCT = 177.26		
		psychophysical problems	1	7.57	0.002	0.46	0.69	Mean of covariate Psychophysical problems = 1.64		
		Double intensification of therapy	1	7.05	0.02	0.44	0.66	Absence Double intensification 13.68 (0.9)		Presence Double intensification 5.10 (2.14)
CBCL externalizing problems	37	Sum of acute complications	1	7.77	0.002	0.46	0.7	Mean of covariate Sum of acute complications = 1.39		

As can be seen from the table, the Total problems were higher in the case of: a female offspring, related allogeneic HSCT, more frequent acute and long-term sequelae, more psychophysical problems, a higher amount of time elapsed between the communication of the need to transplant and the actual transplantation. Externalizing problems, in a similar way to what happens with aggressiveness, were predicted by the presence of more acute consequences. Regarding Internalizing problems, the predictive risk factors were: allogeneic familiar transplantation, a higher number of acute consequences, less time elapsed between the communication of the need to transplant and the actual transplantation, a higher number of psychophysical problems reported, the presence of double intensification therapy.

Table 6 shows the significantly predictive demographic and medical factors on the CBCL syndrome scales.

**Table 6:** **Socio-demographic and medical factors highlighted by ANCOVAs as predictors of the YSR/11–18**.

**Dependent variable**	***N***	***IV***	***df***	***F***	***p***	**η_*p*_*2***	***B***	**Estimated marginal means (*****SD*****)**		
Anxious/depressed	37	Days of hospitalization in Transplantation Unit	1	6.91	0.003	0.43	0.65	Mean of covariate Days of hospitalization in Transplant Unit = 52.35		
		Sum of acute complications	1	18.58	0.002	0.67	0.97	Mean of covariate Sum of acute complications = 1.39		
		Time elapsed between the communication of the HSCT need and the effective HSCT	1	6.34	0.03	0.14	0.61	Mean of covariate Time elapsed between the communication of the HSCT need and the effective HSCT = 177.26		
		Psychophysical problems	1	14.66	0.004	0.62	0.91	Mean of covariate Psychophysical problems = 1.64		
		Gender	1	4.63	0.05	0.34	0.48	Female 6.68 (1.08)		Male 2.43 (0.76)
		Double intensification of therapy	1	4.92	0.05	0.35	0.51	Absence double intensification 5.30 (0.73)		Presence double intensification 1.41 (1.74)
Depression	37	Sum of acute complications	1	13.86	0.005	0.60	0.91	Mean of covariate Sum of acute complications = 1.39		
		Time elapsed between the communication of the HSCT need and the effective HSCT	1	18.21	0.002	0.67	0.96	Mean of covariate Time elapsed between the communication of the HSCT need and the effective HSCT = 177.26		
Somatic	37	GVHD	1	5.71	0.04	0.36	0.58	Absence GVHD 4.20 (0.47)		Presence GVHD 0.85 (2)
		Type of HSCT	2	4.14	0.04	0.45	0.59	Autologous 3.26 (0.87)	Unrelated donor allogeneic 1.87 (0.76)	Related donor allogeneic 6.47 (1.13)
		Current age at the assessment time	1	4.82	0.05	0.32	0.51	Mean of covariate currant age at the assessment time = 5.535.43 days		
Social	37	Double intensification therapy	1	7.47	0.02	0.45	0.68	Absence double intensification 2.47 (0.54)		Presence double intensification 5.93 (1.28)
		Days of hospitalizations post HSCT	1	7.08	0.02	0.44	0.66	Mean of covariate days of hospitalization post-HSCT = 13.42		
		Time elapsed between the communication of the HSCT need and the effective HSCT	1	8.75	0.01	0.49	0.75	Mean of covariate Time elapsed between the communication of the HSCT need and the effective HSCT = 177.26		
Attention	37	Sum of acute complications	1	10.82	0.009	0.54	0.83	Mean of covariate Sum of acute complications = 1.39		
		Time elapsed between the communication of the HSCT need and the effective HSCT	1	5.66	0.04	0.38	0.56	Mean of covariate Time elapsed between the communication of the HSCT need and the effective HSCT = 177.26		
Aggressiveness	37	Sum of acute complications	1	8,66	0.01	0.49	0.74	Mean of covariate Sum of acute complications = 1.39		

Anxiety symptoms were more frequent if the ex-patients: were female, they spent longer time in the Transplantation Unit, showed more acute consequences, were not exposed to double intensification therapy, had a shorter time occurred between the communication of the HSCT need and the actual HSCT, reported higher psychophysical problems.

Depressive symptoms were more evident in the case of a higher presence of acute consequences and of a higher latency between the communication of the need to transplant and the actual transplantation.

The Somatic complaint scale was influenced by: absence of GvHD, allogeneic familiar transplantation and younger age at assessment.

Social symptoms were predicted by a longer hospitalization after transplantation, the presence of double intensification therapy during treatment, and where a higher amount of time had elapsed between the communication of the need to transplant and the actual transplantation.

Attention symptoms were shown to be negatively influenced by acute consequences and by a longer time elapsed between the communication of the need to transplant and the actual transplantation.

Subsequently, in order to investigate whether specific demographic or medical aspects could influence behavioral/emotional problems as reported by the HSCT survivors in the YSR, hierarchical linear regression analyses (HLM) were run inserting as dependent variables in the model the YSR scales and the following variables as independent ones: age at transplantation, amount of time elapsed from HSCT, amount of time elapsed from the communication of the need to transplant and the actual transplantation, days of hospitalization in the Transplantation Unit, sum of acute complications, sum of chronic complications, sum of psychophysical problems. The first block included socio-demographic variables and objective medical variables (age at transplantation, time elapsed between transplantation and assessment, days of hospitalization in the Transplantation Unit, time elapsed between the communication of the need to transplant and the effective transplantation) whereas the second block included variables referring to the psychological and physical consequences of transplantation (acute and long-term complications, sum of psychophysical problems). The results of HLM with depressive symptoms as a dependent variable highlighted that the second model explained the majority of variance (*R*^2^ = 0.23) with amount of time passed from transplantation to the assessment (ß = 0.58, *p* = 0.03), the only factor that impacted significantly on depressive symptoms.

HLM with social problems as a dependent variable showed the third model as the best one (*R*^2^ = 0.18; *p* = 0.01) where these problems were significantly influenced by a higher presence of psychophysical problems (ß = 0.80, *p* = 0.015) and days of hospitalization post HSCT (ß = −1.15, *p* = 0.05).

## Discussion

The aim of the present study was to investigate the psychological aspects associated with HSCT in pediatric age. Although HSCT increases the possibility to survive and to recover from disease it affects all aspects of the child's and the adolescent's life, including social, cognitive, emotional and behavioral functioning. More specifically, the objectives of the study were to: (a) assess the presence of psychopathology in HSCT survivors at distance from transplantation, (b) compare the psychological aspects of pediatric HSCT survivors with those of healthy peers, (c) detect whether parents and children differed in the perception of mental health status, and (d) identify medical and socio-demographic factors associated with psychopathology. For these purposes, the CBCL and the YSR were given to a group of HSCT survivors and their families and to a comparison group matched according to age, gender and geographic origin.

With respect to the first objective (a), both the parents and the survivors reported scores above normative cut offs for the *Total competence scale*, highlighting the presence of difficulties in the domains concerning activities and social competencies. These results seem to be in line with the hypothesis made by some authors that the experiences associated with transplantation could somehow interfere with adjustment and social competence (Phipps and Mulhern, 1995; Vannatta et al., 1998). Regarding more specifically the presence of psychopathology, on the other hand, higher scores were reported for somatic symptoms, suggesting both the presence of actual long-term somatic sequelae after treatment or a possible decrease in pain tolerance, as already highlighted by the literature (Pederson et al., 2000).

It is interesting to note that, in contrast to what other studies reported (Kazak et al., 2001, 2004), the group of subject involved in our study did not report significant scores in the *Post-traumatic stress problems* scale. It is possible to speculate as to whether HSCT survivors could experience anticipatory fear linked to their previous traumatic experience and express it through worries about physical well-being, a hypothesis that could in part explain the parallel presence of high scores on somatic symptoms and low scores on post-traumatic stress symptoms (Bingen et al., 2012; Ahomäki et al., 2015).

With reference to the second objective (b), the comparison between the HSCT Group and the HEALTH Group highlighted differences only regarding somatic symptoms. No differences were found with respect to the other variables. This is not surprising, given the high probability that the majority of survivors has to experience physical late effects after transplantation (Skinner and Leiper, 2004). Moreover, this data should also be interpreted considering the fundamental role played by the body both during childhood and adolescence. As previously highlighted childhood represents a developmental period characterized by a close relationship between body and mind, and where emotional difficulties are likely to be expressed through somatic complaints. On the other hand, during adolescence bodily issues are of particular importance especially given the processes of physical and sexual growth, which are typical of this period.

With respect to the third objective (c), the comparison between the CBCL and the YRS reported low inter-rater agreement only in a few scales (i.e., *Total competence, somatic problems and attention problems*), suggesting a different perception of psychopathology between the survivors and their parents. More specifically, the results showed higher scores on the CBCL, suggesting the perception of higher behavioral/emotional difficulties on the parents' side. The low agreement between child and parent reports has already been described by other studies (Perrin et al., 1991; Briggs-Gowan et al., 1996). This data could be read both as a higher denial of psychopathology in the survivors but also as their possibility to reach a more functional adjustment, an achievement that could be somehow favored by their previous experience. Moreover, some authors have suggested that the parents' emotional state might influence their perception regarding the children/adolescent's emotional state in a way that, for example, a higher degree of anxiety experienced by caregivers might lead to a higher parental perception of anxiety in the adolescent as well (Parsons et al., 2006, 2009; Jobe-Shields et al., 2009; Chang et al., 2012). In this sense, the collection of multiple perspectives would be preferable during the assessment of HSCT survivors' well-being, in order to better identify subjects that are potentially at higher risk of psychological problems and to promptly offer supportive interventions (Chang et al., 2012).

Finally, with respect to the fourth objective (d), the results evidenced an association between specific medical and socio-demographic variables and the risk of experiencing higher psychological distress. More specifically, with respect to the *Total problem scale* and to the summary scales, the variables female gender, related allogeneic HSCT, acute and long-term sequelae, psychophysical problems, and time elapsed between the communication of the need to transplant and the effective transplantation were shown to be predictors of a higher risk of experiencing psychopathology. These results are in line with the literature that identifies females at a higher risk of developing psychopathological problems subsequent to oncologic disease and transplantation (Ahomäki et al., 2015). Furthermore, allogeneic related transplantation was shown to be a risk factor for experiencing a higher degree of emotional and behavioral difficulties, in accordance with other studies that already identified children undergoing autologous transplant as less likely to experience significant somatic distress and impairments when compared with those exposed to allogeneic intervention, both related or unrelated (Phipps et al., 2002). On the other hand, it is possible to speculate that although the presence of a related donor could represent a precious resource in terms of survival, it may increase the possibility of experiencing intense psychological distress in the patient (Weaver et al., 2015). Moreover, the physical suffering associated with medical toxicities and waiting, before the actual transplantation after the communication of the need to transplant, that could be either too long or too short, could constitute additional psychological stressors.

It is interesting to observe that the presence of acute consequences predicted higher externalizing symptoms, which could thus be interpreted as a possible reaction to the “internal,” physical and psychological sufferings linked to disease and transplantation.

On the other hand, regarding the syndrome scales, again female survivors were identified at higher risk of experiencing anxiety. Anxiety symptoms were also predicted by a higher amount of time passed in the Transplantation Unit, by the presence of acute consequences and psychophysical problems, and by a shorter time elapsed between the communication of the need to transplant and the actual transplantation. On the contrary, a longer amount of time passed before the intervention predicted depressive symptoms, suggesting the need to schedule the “right” timing between the different phases that follow diagnosis, in order to favor a more functional regulation of the emotional aspects involved in this delicate process.

Moreover, somatic complaints were predicted by a younger age at the moment of diagnosis. This result, that seems somehow to contrast with other studies that suggest older age to be a significant variable associated with poorer psychological functioning (Parsons et al., 2006), could be interpreted with specific reference to the developmental phase that each patient is experiencing; while children could be more likely to complain about somatic disease and to ask their caregivers for help, it is plausible that in adolescence, a period specifically characterized by the search for autonomy, the individuals are more inclined to deal with the difficulties by themselves. Social problems were predicted by a longer time elapsed between the communication of the need to transplant and the actual transplantation, by the presence of longer hospitalizations, and by the presence of intensification with double HSCT, suggesting that the concrete limitations in activities subsequent to medical procedures that patients experience could significantly impact on their relationships with peers. Finally, the variables considered also predicted attention symptoms, in line with the studies that pointed out the possible impact that hospitalizations and medical procedures may have on the cognitive functioning of psychiatric patients (Bingen et al., 2012).

One limitation of the present study concerns the small number of participants, which prevents the generalization of the data obtained; in any case, these results seem to be in line with the existing literature in confirming the presence of an increased risk of experiencing psychological distress and psychopathology in ex-patients exposed to HSCT during childhood. This risk seems to be enhanced by the presence of specific demographic and medical variables, which should thus be taken into account during the different phases that precede, accompany, and follow HSCT. Taken together, these results could help to inform intervention and prevention programs, since attention to the aforementioned demographic, medical and psychological variables could help identify which subjects could still require services and support once the process of reintegration to normal routines following HSCT has begun.

## Author contributions

SZ: wrote the central part of the manuscript, partecipated in collecting data. AT: wrote the discussions section of the manuscript; partecipated in critical interpretation of data. MTr: prepared data set, performed statistical analyses. FS: prepared data set, performed statistical analyses. AP: wrote the introduction section of the manuscript and participated to the organization of collected data. GP: performed analyses and supervised statistical analyses. ND: prepared tables and figures; recruited the sample and wrote the references. FC: recruited the sample and followed patients in every steps of the project. AM: recruited the sample and collected medical data; carried out follow up visits. MTu: recruited the sample and collected medical data. CC: prepared the study design and supervised the mental health research team. GB: prepared the study design and supervised the research team. CM: prepared the study design and supervised the research team. All authors reviewed the manuscript.

### Conflict of interest statement

The authors declare that the research was conducted in the absence of any commercial or financial relationships that could be construed as a potential conflict of interest.

## Footnotes

^1^Regarding medical variables, data concerning anamnesis and the treatment process were collected through a specific checklist that investigated: (a) duration of hospitalization in Transplantation Unit, duration of hospitalization post-transplantation, time elapsed from the communication of the need to transplant and the actual transplantation, (b) admission to Pediatric Intensive Care Unit (PICU), (c) presence of acute or chronic medical complications (toxicity), (d) presence of Graft vs. Host Disease (GvHD). A severity index was attributed to each acute complication/toxicity, according to the Common Terminology Criteria for Adverse Events (CTCAE). The CTCAE scale provides a grading (severity) to each adverse event (AE): 0 = absence of side effects, 1 = mild AE, 2 = moderate AE, 3 = severe, medically significant and unexpected AE, 4 = severe, disabling, and life-threatening AE, 5 = lethal AE. Subsequently, the organs affected by grades 3,4, and 5 were taken into account.
